# Donor−acceptor−donor (D-A-D) structural monomers as donor materials in polymer solar cells: a DFT/TDDFT approach

**DOI:** 10.1080/15685551.2021.1997178

**Published:** 2021-11-08

**Authors:** Numbury Surendra Babu

**Affiliations:** Computational Quantum Chemistry Lab, Department of Chemistry, College of Natural and Mathematical Sciences, the University of Dodoma, Dodoma, Tanzania

**Keywords:** 3, 6-carbazole, D-A-D monomers, DFT/TD-DFT method, electronic properties, optoelectronic properties

## Abstract

Density functional theory (DFT) and time-dependent DFT (TD-DFT) are used to investigate the ground- and excited-state properties of donor-acceptor–donor (D-A-D) monomers based on 3,6-carbazole (CB) combined with various-conjugated benzothiazole derivatives, using B3LYP and the 6–311 G basis set. To create nine D-A-D monomers for this investigation, nine (9) distinct acceptors were inserted at the C3 and C6 positions of carbazole. The impact of various electron-donor groups on structural, electrical, and optoelectronic properties is investigated. Our technique for developing novel donor monomers provides a theoretical framework for further optimizing the photovoltaic device’s electrical, optical, and efficiency features. The HOMO and LUMO energies, bandgap, excited state, exciton binding energy, open-circuit voltage (V_OC_) and absorption spectra were calculated. Our findings indicate that CB-TDP-CB and CB-SDP-CB monomers have an appropriate electronic structure for polymer solar cells.

## 1. Introduction

Solar energy collecting is one of the most challenging issues facing today’s renewable energy specialists. Researchers are working hard to develop low-cost, dependable, elastic, and ecologically friendly optoelectronic devices with high efficiency [1–4]. Although silicon-based materials are appropriate for current solar cell technology, their high cost and high operating temperature limit their portability and adaptability [5]. Conjugated polymers (CPs) were used as a step toward the creation of low-cost, environmentally friendly, easily synthesizable, flexible, and efficient materials for solar cells [4,6]. Because of their stability, low-cost manufacturing, and ability to build tunable and robust structures, CPs is emerging as attractive materials. Scientists are investigating four prominent generations of CPs, which have applications in solar to electrical energy conversion [7–11].

Conjugated polymers have sparked significant interest in recent decades [12,13] due to their potential applications in fields such as organic field-effect transistors [14], organic light-emitting diodes [15], electrochromic devices [16], photovoltaics [17], electronic displays, supercapacitors, thermoelectric devices [18], and so on.

Conjugated polymers with a donor-acceptor donor (D-A-D) backbone are promising candidates for organic semiconductors because they allow for the customization of optoelectronic properties by modifying the donor and acceptor units. A wide range of electron-rich and electron-deficient polymers can be synthesized by carefully selecting donor and acceptor units, resulting in a wide range of electron-rich and electron-deficient polymers for electron and hole stabilization with fine bandgap control and energy levels as needed. The copolymerization of D-A-D monomer with other building blocks can more effectively modify the optoelectronic properties of D-A-D polymer. Copolymers have recently gained much attention because of their tunable physical and chemical properties, such as bandgap, highest occupied molecular orbital (HOMO), lowest unoccupied molecular orbital (LUMO), optical absorption, shape, solubility, stability, and so on [19]. It should also be mentioned that a wide range of donor and acceptor units are now available for fine-tuning the optoelectronic properties [20].

D-A-D plays a vital function in charge separation and molecular architecture, which regulates charge transfer. The D-A-D system exhibits reduced band gap, significant charge transformation, and improved visible light absorption due to considerable overlapping of D and A molecular orbitals. Therefore, one of the criteria for generating a large photocurrent has been the synthesis of donor and acceptor materials with complementary absorption characteristics intended to increase the coverage of the solar spectrum. As a result, for OPVs, a range of narrow optical gap non-fullerene acceptors (NFAs) based on a stronger electron-donating core were carefully designed and synthesized [21]. Furthermore, utilizing a D-A-D fused core can choose a somewhat planar structure rather than a twisted one, facilitating electron flow from donor to acceptor since the planarized D-A-D promotes -electrons delocalization. More crucially, a modest electron perturbation may occur in the D-A-D fused core, resulting in efficient charge transfer [22]. In other words, when the triazole is introduced into this fused system, -orbital electrons are less likely to be caught while travelling through the fused -bridge [23].

The current study’s novelty stems from the usage of new donor-acceptor-donor monomers as a 3,6- carbazole donor and benzothiazole-based derivates as acceptors, based on these preliminary findings and theoretical models. Numerous studies show alternating polymeric architectures to identify the best poly(3,6-carbazole) derivatives for BHJ solar cell devices [24–27]. Quantum calculations were performed on the repeat unit of the proposed polymers to determine both the HOMO and LUMO energy levels to evaluate the poly-carbazole performances utilizing those models. These novel polymers were put through their paces in solar cell devices, and their performance was assessed in terms of polymer organization, molecular weight, charge carrier mobility, and LUMO energy level.

Carbazol-based polymers (PCz) have gotten much interest in the last 50 years because they are more stable and have a more significant redox potential than other conducting polymers [28]. Similarly, their excellent hole transportation mobility and substantial absorption in the UV spectral region have good electro- and photoactive characteristics [29]. It is undeniably a successful method to raise the HOMO level while decreasing the optical gap. Standard building blocks for boosting a single component’s photoluminescence quantum yield (PLQY) are benzothiazole-based conjugated compounds with distinctive luminescence characteristics [30]. Furthermore, the strong PLQY indicates efficient radioactive recombination pathways, leading to a high electroluminescence yield of the final devices. As a result, it aims to insert benzothiazole into the central core to construct an electron-deficient-core-based fused structure (D-A-D) for altering the resulting molecules’ optoelectronic properties to achieve low voltage loss and high device performance.

**Scheme 1.**
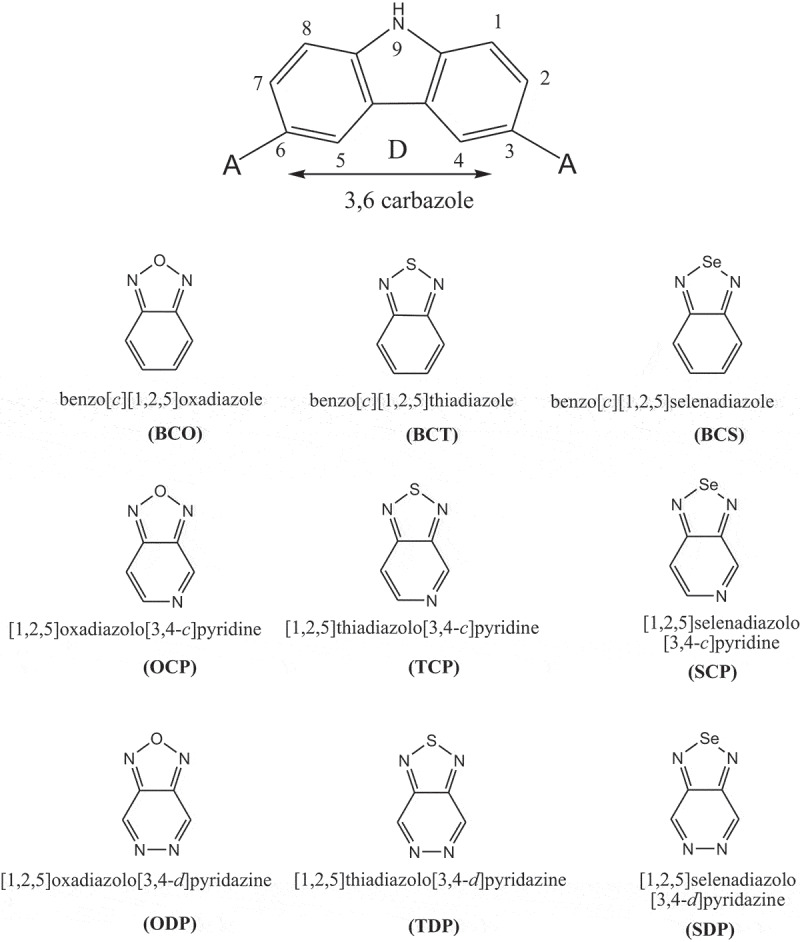
Building units as donor/acceptor moieties

In this investigation, D-A-D monomers 3,6-carbazoles were utilized as donors (D) and accepters such as: benzо[с] [1,2,5] оxаdiаzоle (BСО); benzо[с] [1,2,5] thiаdiаzоle (BСT); benzо[с][1,2,5]selenаdiаzоle (BСS); [1,2,5] оxаdiаzоlо[3,4-с]рyridine (ОСР); [1,2,5] thiаdiаzоlо [3,4-с]рyridine (TСР); [1,2,5] selenаdiаzоlо [3,4-с] рyridine (SСР); [1,2,5] оxаdiаzоlо[3,4-d]рyridаzine (ОDР); [1,2,5] thiаdiаzоlо[3,4-d]рyridаzine (TDР) аnd [1,2,5]selenаdiаzоlо[3,4-d]рyridаzine (SDР) (Scheme 1).

Because of high-performance computing and optimization of computational chemistry programs, theoretical investigations in this area have increased in recent years. The theoretical method is the most effective instrument for overcoming difficulties in experimental synthesis and exploring alternatives that lower material production and processing costs. Because DFT investigates this type of material’s electronic structure and spectroscopic properties, it represents a reliable alternative for tackling these jobs. We created a new D-A-D type of small-molecule OPV donor in this study. Because there are so many conceivable D and A unit combinations, a virtual pre-synthesis screening would be required for a logical design of small-molecule OPV donors. With density functional theory (DFT) and time-dependent DFT (TDDFT) calculations at the B3LYP/6-31 G level, we have well defined molecular orbital (MO) energy levels, bandgaps, UV/VIS absorption spectra, and PCEs of diverse D-A-D polymer donors.

## 2. Computational calculation details

The same calculation method as in our prior investigations [31,32] is used. The DFT approach of Becke’s three-parameter compound (B3LYP) [33–35] was employed in all of the neutral monomer studies in this work. All computations were performed using the 6–311 G basis set. The Gaussian09 package [36] was used to perform the density (DFT) and time-dependent (TD-DFT) functional calculations [37]. We determined the HOMO and LUMO energies of the molecules analyzed using optimal structures; we also determined the bandgap energy (Eg), which was calculated as the difference between the HOMO and LUMO energy levels. At the TD-DFT/B3LYP/6-31 G level, the absorption spectra and excited states of all the monomers (D-A-D) structures were computed. The Gausssum software tool was also used to visualize the total density of states (TDOS).

Because most chemical activities occur in the solution phase, solvent effects garner a lot of interest. Therefore, the solvent effects are included here to guarantee that the calculations are compatible with the common experimental settings. The following section goes into greater detail about the impact of the chemical environment. To investigate the effect of solvents on ground-state molecular geometry, quantum chemical calculations of excitation energies and absorption maxima on the examined molecules were performed in both vacuum and solvent. In the TD-DFT calculations, chlorobenzene was employed as the solvent within the polarizable continuum model (PCM) [38].

## 3. Results and discussions

### 3.1. Molecular design and geometry structures

**Figure 1.**
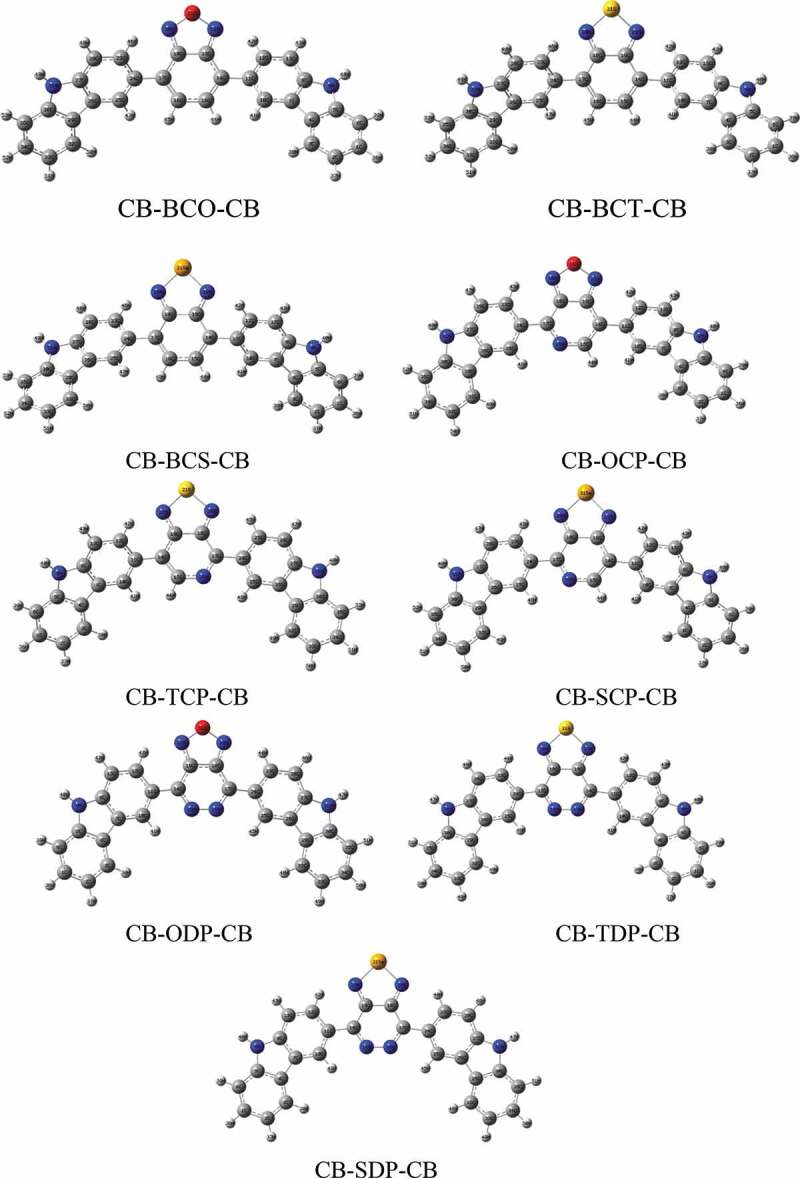
Optimized geometries of the studied molecules obtained by B3LYP/6-311 G in the gas phase

The structural properties of the nine D-A-D monomers examined are depicted in Fig. S1, and their optimal geometries are displayed in Figure 1. The molecules are completely optimized in the neutral states to establish the geometric parameters; we used the 6–311 G basis set. We found that the geometric characteristics are slightly affected by the different acceptor groups connected to the donor molecule. Table 1 shows the inter-cyclic lengths and dihedral angles between D-A in D-A-D monomer determined from the optimized structures of the examined molecules in the neutral state in gas and solvent. Planarity in molecule geometry and the corresponding -electrons conjugation over the backbone influence a chemical substance’s visible light absorption. An ideal 1800 dihedral of the D-A-D (Table 1) demonstrates that the combination of donor and acceptor moieties planarized the geometry of the resultant polymer by producing a delocalized -electronic cloud density across the backbone. The D-A-intra-chain D’s dihedral angle (1800) is responsible for its low cost and simple synthesis technique.

**Figure 2.**
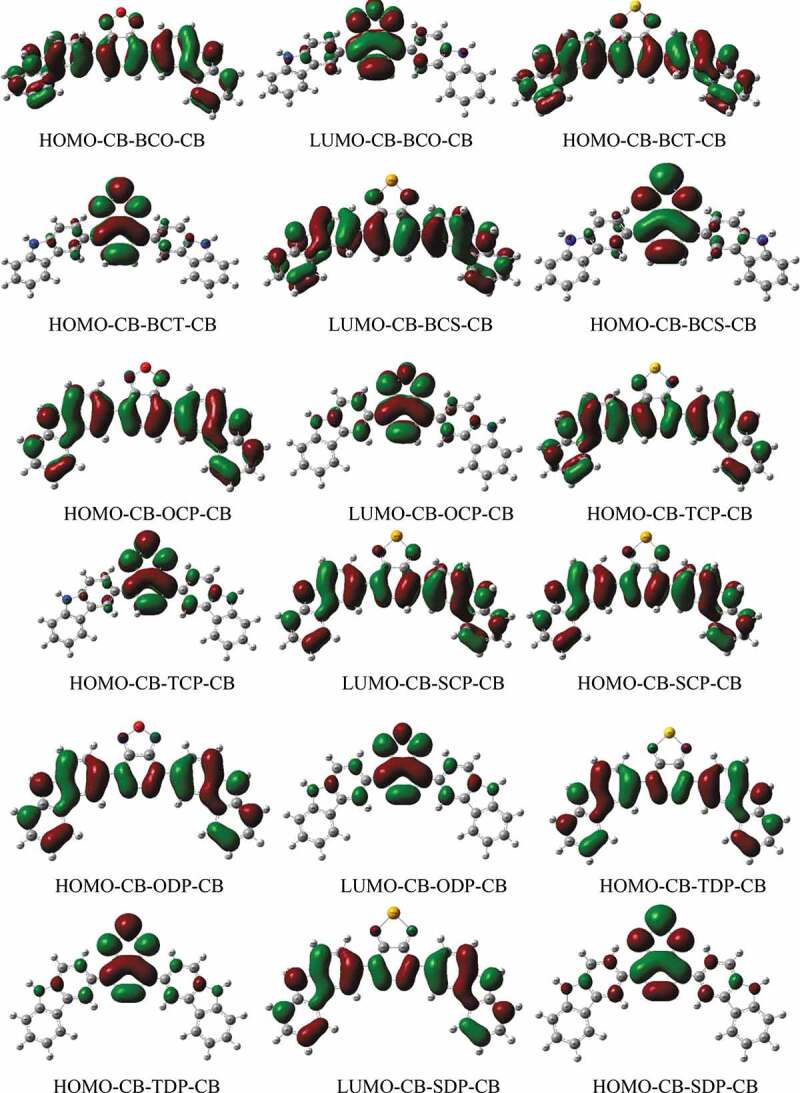
Representation of the frontier molecular orbitals (HOMO and LUMO) obtained from DFT//B3LYP/6-31 G+(d,p) calculations in the gas phase

**Figure 3.**
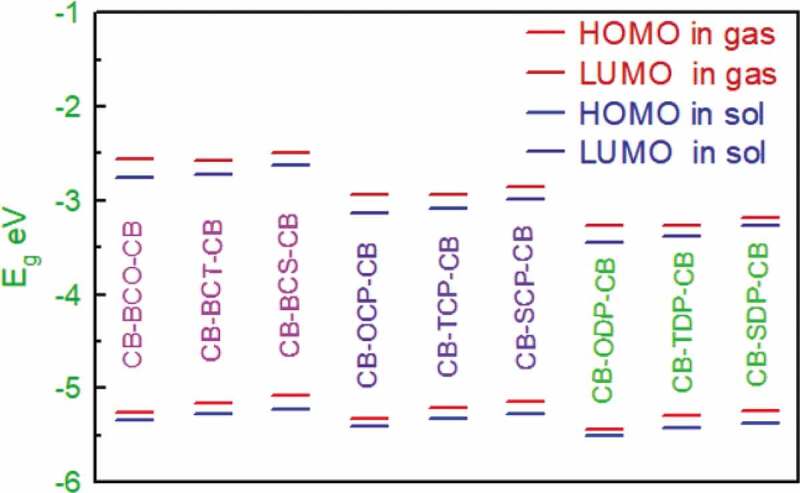
The HOMO and LUMO energy levels of the studied D-A-D monomers at DFT/ B3LYPlevel with 6–311 G basis set

**Figure 4.**
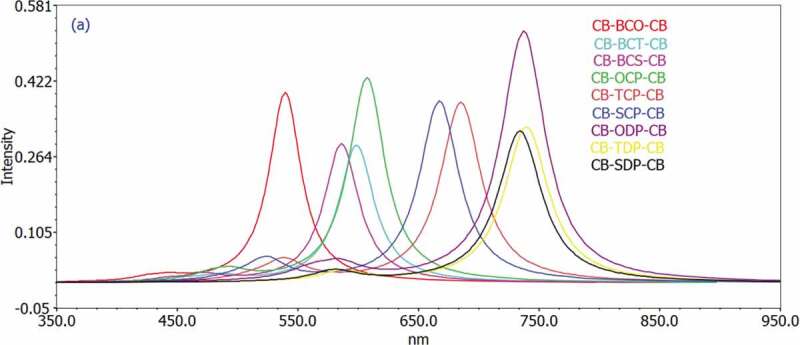
Simulated UV-Visible optical absorption spectra of the studied carbazole copolymer monomers (D-A-D) calculated by TD/DFT/ B3LYP/6-311 G level in the gas phase

**Figure 5.**
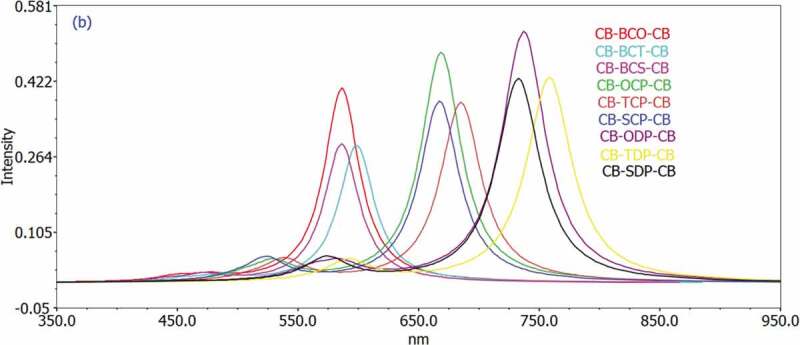
Simulated UV-Visible optical absorption spectra of the studied carbazole copolymer monomers (D-A-D) calculated by TD/DFT/ B3LYP/6-311 G level in the solvent phase

**Figure 6.**
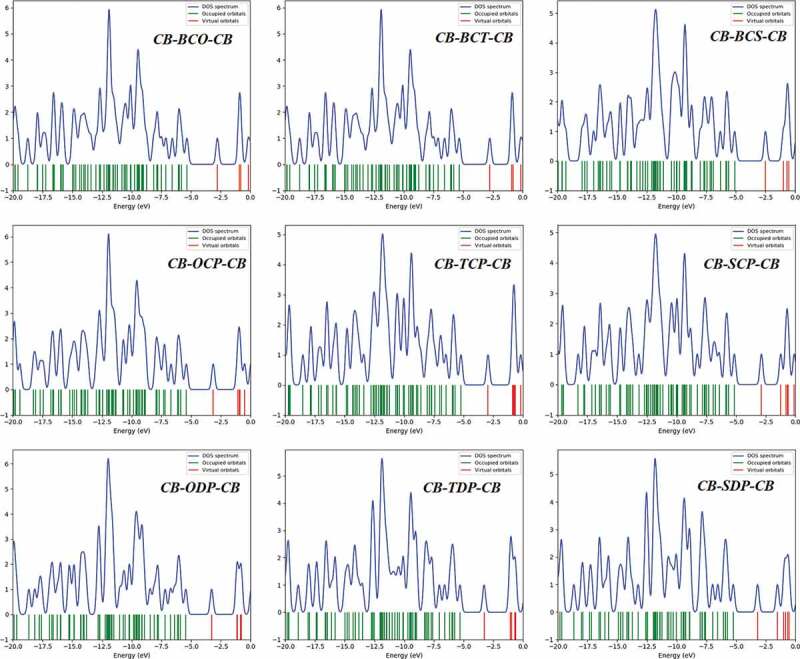
The density of states (DOS) diagram produced by GaussSum for Studied D-A-D monomers

**Table 1.** Intercyclic distances (Å) and dihedral angles between D-A unit’s values of the studied monomers obtained in the gas and solvent phase by using DFT/B3LYP with 6–311 G basis set

		gas	chlorobenzene	gas	chlorobenzene	gas	chlorobenzene
S.No	D-A-D	D-A(Å)	D-A(Å)	Dihedral (θ°)	Dihedral (θ°)	μ	μ
1	CB-BCO-CB	1.47840	1.47860	30.35	32.13	3.0611	4.0382
2	CB-BCT-CB	1.48140	1.48172	35.95	38.34	0.9110	1.3474
3	CB-BCS-CB	1.48173	1.48203	34.56	37.43	0.2879	0.2266
4	CB-OCP-CB	1.46779	1.46744	0.0	0.0	1.7191	2.0956
5	CB-TCP-CB	1.47762	1.47776	−33.90	−36.21	0.8550	1.1914
6	CB-SCP-CB	1.47423	1.47540	0.0	0.0	1.9825	2.7449
7	CB-ODP-CB	1.46645	1.46542	0.0	0.0	0.1593	0.1419
8	CB-TDP-CB	1.47188	1.47213	0.0	0.0	2.2478	3.2962
9	CB-SDP-CB	1.47261	1.47333	0.0	0.0	3.5359	5.0814

According to the findings in Table 1, the dihedral angles in the intramolecular rings of D-A-D are likewise close to planarity. We discovered a modest effect of the acceptor group on the inter-cyclic distance, but the impact on the dihedral angles θi° (i = 1–9) is visible in both the gas and solvent phases. We also said that the solvent phase affects these factors. In reality, the inter-cyclic lengths decrease while the dihedral angles increase significantly, allowing for improved electrical conjugation. In the monomer models, the two units are twisted from each other in the minimum energy structures of 1, 2, and 5 by 300–380, most likely due to repulsion between the two hydrogen atoms projecting from the phenyl groups present in both units. The repulsion was removed by substituting a benzothiazole for the phenyl group in the fused-ring unit; hence, 4,6,7,8 and 9 have almost planar lowest energy structures.

### 3.2. Electronic prosperities

#### 3.2.1. Frontier molecular orbitals (FMOs)

FMOs are one of the most underlying molecular orbitals because of their association with chemical species reactivity. The FMOs in PSCs are critical for understanding and predicting how charge transfer throughout the polymer occurs due to an excitation event. The HOMO and LUMO diagrams of the D-A-D monomers are shown in Figure 2. The HOMO density is spread along with the structure for all D-A-D monomers, as seen in Figure 2. Furthermore, this FMO is found in the C-C bonds of the carbazole donor group. The density of the LUMO, on the other hand, is primarily distributed among the acceptor groups. As a result of their excellent superposition, the current PSC candidates provide an ideal conduit for electron transport from the HOMO to LUMO orbital.

Predicting the behaviour of new polymers’ HOMO and LUMO energy levels is critical for rationally designing optimum BHJ solar cells. We computed the HOMO and LUMO energy levels of the D-A-D monomers using density functional theory (DFT) as represented by the B3LYP functional and the 6–311 G basis set in this work. The DFT/B3LYP/6-311 G formalism has been proven to be an accurate framework for determining numerous molecular systems’ structural and optical properties [39]. The HOMO and LUMO energy levels and the bandgaps of the copolymers are directly related to the efficiency of solar cells.

The electrical properties were utilized to anticipate the primary qualities useful in polymer solar cells. Table 2 shows some of the parameters found via DFT calculations. Figure 3 depicts the FMO energy levels and energy gaps of the all monomers model (n = 1–9). In addition, the HOMO energy levels of the 1–9 monomers in the gas phase and solvent phase are presented in Table 2, implying that these systems may have strong air stability when applied to BHJSC with PC60BM. Indeed, a statistical review [40] proposes that within a photovoltaic device employing PC60BM as an acceptor, a lower HOMO level (closer to vacuum) of the polymer donor will aid improve open-circuit voltage (V_OC_) devices.

**Table 2.** Calculated E_HOMO_, E_LUMO_ levels, energy gap (E_g_) values of the studied monomers obtained by DFT/B3LYP/6-311 G G level

S.No	monomer	gas		chlorobenzene		gas	chlorobenzene
	D-A-D	HOMO eV	LUMO eV	HOMO eV	LUMO eV	E_g_	E_g_
1	CB-BCO-CB	−5.2574	−2.5559	−5.3339	−2.7620	2.7015	2.5719
2	CB-BCT-CB	−5.1649	−2.5674	−5.2784	−2.7282	2.5975	2.5502
3	CB-BCS-CB	−5.0857	−2.4904	−5.2202	−2.6245	2.5953	2.5957
4	CB-OCP-CB	−5.3328	−2.9339	−5.3992	−3.1333	2.3989	2.2659
5	CB-TCP-CB	−5.2147	−2.9404	−5.3249	−3.0838	2.2743	2.2411
6	CB-SCP-CB	−5.1407	−2.8606	−5.2729	−2.9799	3.7448	2.2930
7	CB-ODP-CB	−5.4408	−3.2637	−5.5083	−3.4422	2.1771	2.0661
8	CB-TDP-CB	−5.2999	−3.2653	−5.4158	−3.3802	2.0346	2.0356
9	CB-SDP-CB	−5.2349	−3.1837	−5.3731	−3.2738	2.0512	2.0993

Table 2 shows the HOMO-LUMO energy gaps (E_g_ = ELUMO – EHOMO) for D-A-D monomers. Except for the CB-SCP-CB monomer, the bandgap (E_g_) values are less than 3 eV. CB-TDP-CB> CB-SDP-CB> CB-ODP-CB> CB-ODP-CB> CB-TCP-CB> CB-OCP-CB is the order of D-A-D monomers concerning E_g_. In both phases, CB-BCS-CB CB-BCT-CB CB-BCO-CB CB-SCP-CB The lower E_g_ of CB-TDP-CB and CB-SDP-CB, compared to other monomers, illustrates the importance of intramolecular charge movement, which causes the ingestion spectra to redshift. This is clearly due to the impact of the electron-contributor unit, which is more solid for 3,6-CB-TDP-CB and 3,6-CB-SDP-CB than for other monomers. Therefore, all atoms with a minimal energy gap must have extraordinary photophysical properties, particularly 3,6-CB-TDP-CB.

This suggests that the degree of inductive effects of the acceptor units considerably influences the band gaps of the constructed molecules. Furthermore, these findings indicated that adding sulfur, nitrogen, and oxygen groups help close energy gaps. In that scenario, the CB-SCP-CB monomer exhibits the most significant energy gap and a drop in the HOMO level, which should be directly related to the increased electron-donating capability of the SCP acceptor unit containing the Se atom. These findings indicate that acceptor units have a significant impact on the LUMO distribution and, as a result, the HOMO-LUMO energy gaps. It is proposed that the disparity in the distribution of the LUMO orbital for the designed systems is due to the considerable variance in their geometric qualities, as 3,6-CB-TDP-CB and 3,6-CB-SDP-CB are the most planar and hence the most conjugated.

### 3.3. Photovoltaic properties

#### 3.3.1. Scharber’s model

The device’s maximum power density divided by the total power density received from the Air Mass 1.5 solar spectrum [41], which is 1000 W/m^2^. The power density of the device is made up of the open-circuit voltage, short-circuit current density, and fill factor (FF). According to Scharber’s model, the V_OC_ is associated with the gap between the acceptor’s LUMO and the donor’s HOMO. Subtracting 0.3 eV from the energy level difference yields the V_OC_. This change was identified empirically and is associated with residual carrier binding energy and interface effects [42]. External quantum efficiency (EQE) multiplied by the number of photons from the Air Mass 1.5 sun spectrum at all frequencies. The EQE is just a step function with a value of 0 per cent for energies below and above the donor’s optical band gap (E_opt_). For all devices, the fill factor is FF = 0.65. If needed, further EQE and FF assumptions can be made. The EQE, for example, could be found by analyzing the Kohn-Sham joint density of states, which reveals the frequency-dependent absorption cross-section behaviour. In this case, the polymer layer is thick enough to absorb any photon passing across the optical band gap, while the film structure effectively restricts the EQE. Scharber’s mode is described by the equations below.

(1) $$PCE=\frac{V_{OC}J_{SC}FF}{1000W/m^{2}}$$

(2) $$LUMO_{donor}>LUMO_{acceptor}+0.3eV$$

(3) $$eV_{OC}=LUMO_{acceptor}-LUMO_{donor}$$

(4) $$E_{opt}=LUMO_{donor}-HOMO_{donor}$$

(5) $$EQE\left(\omega \right)=0.65\times \Theta \left(\hbar \omega -E_{opt}\right)$$

(6) $$J_{SC}=\int EQE\left(\omega \right)\times \#photons_{AirMass1.5}\left(\omega \right)d\omega$$

This model requires a 0.3 eV energy gap between the donor and acceptor Lumos to achieve successful charge transfer, as shown in eq 4. However, this LUMO offset should not be confused with the empirical shifts of 0.3 eV for eq 5. As a result, the highest value for eV_OC_ is Eopt = 0.6 eV.

The LUMO level prediction is not as precise as the HOMO level prediction may potentially be a concern; nonetheless, DFT energy levels must be evaluated in the context of the resulting quantities of interest. According to eqs 4, 2, and 3, the LUMO level allows us to determine the optical band gap, LUMO offset, and open-circuit voltage. The optical band gap description is irrelevant in this scenario because the LUMO level description has been fitted to the bandgap. Because the LUMO offset criteria are required to enable a functional device, donor polymers with LUMO values close to the 0.3 eV limit of eq 2 may encounter difficulties. A higher level of uncertainty in the LUMO level value, on the other hand, is not an issue for values that are far from the offset limit.

In general, the optimization of polymer-fullerene solar cells is based on fine-tuning the electronic properties and interactions of the donor and acceptor components in order to absorb the most light and generate the most free charges while consuming the least amount of energy and transporting the charges to the electrodes. However, in order to develop next-generation, high-efficiency solar systems, it is vital to understand the optimal electronic characteristics that each component should have. For a variety of reasons, fullerenes are now regarded as the best acceptors for organic solar cells. For starters, they contain an energetically deep-lying LUMO, which gives the molecule an extremely high electron affinity in comparison to the numerous possible organic donors [43].

Except for charge collection, which is based on the electrical contact between the active layer composite and the appropriate electrode, it is clear that the active layer donor-acceptor composite now regulates all aspects of the mechanism. The energy difference between the donor’s HOMO and the acceptor’s LUMO is shown to be extremely closely connected with the V_OC_ value [6,44,45]. Therefore, the maximal open-circuit voltage (V_OC_) of the BHJ solar cell is proportional to the difference between the electron donor’s highest occupied molecular orbital (HOMO) and the electron acceptor’s LUMO, after accounting for energy lost during photo charge creation [46]. The following expression was used to compute the theoretical values of open-circuit voltage V_OC_:

(7) $$V_{OC}=\frac{1}{e}\left(\left|E_{HOMO}^{Donor}\right|-\left|E_{LUMO}^{PCBM}\right|\right)-0.3$$

All donors in this investigation were computed using the [6,6]-phenyl-C_60_-butyric acid methyl ester (PC_60_BM) fullerene derivative as the electron acceptor and the VOC values are shown in Table 3. These values are adequate for an efficient electron injection system. As a result, all compounds investigated can be employed as organic solar cell components since electron injection from the excited molecule to the acceptor’s conduction band (PC_60_BM), and subsequent regeneration is achievable in photovoltaic cells.

**Table 3.** The open circuit voltage V_ОС_ (eV) and First singlet excitation energy (E_oрt_), exciton binding energy (E_B_) of the studied D-A monomers

Monomer	V_OC_ (eV)/ PC_60_BM		E_OPT_		E_B_	
	Gas	chlorobenzene	gas	chlorobenzene	gas	chlorobenzene
CB-BCO-CB	0.6574	0.7339	2.2977	2.1139	0.4038	0.4580
CB-BCT-CB	0.5649	0.6784	2.1529	2.0705	0.4446	0.4797
CB-BCS-CB	0.4857	0.6202	2.1486	2.1145	0.4467	0.4812
CB-OCP-CB	0.7328	0.7992	2.0414	1.8537	0.3575	0.4122
CB-TCP-CB	0.6147	0.7249	1.8853	1.8092	0.3890	0.4319
CB-SCP-CB	0.5407	0.6729	1.8869	1.8576	1.8579	0.4354
CB-ODP-CB	0.8408	0.9083	1.8429	1.6819	0.3342	0.3842
CB-TDP-CB	0.6999	0.8158	1.6762	1.6343	0.3584	0.4013
CB-SDP-CB	0.6349	0.7731	1.6884	1.6915	0.3628	0.4078

### 3.4. Optoelectronic parameters

#### 3.4.1. Electronic absorption spectra

The excitation energy based on the first and second singlet-singlet electronic transitions has been examined to understand the electronic characteristics of D-A-D monomers better. Time-dependent density functional theory (TD-DFT) has evolved as a trustworthy standard method for the theoretical study of electronic excitation spectra in recent years, with recent publications demonstrating improved accuracy for a wide range of systems [47,48]. Based on the improved geometry, the TD-DFT/B3LYP/6-311 G was employed. Tables 4 and Tables 5 show the nature and energy of the electronic transitions of all monomers in all series under investigation in the gas and solvent phases. All electronic changes are type, and there are no localized electronic transitions among the estimated singlet-singlet transitions. In addition, Tables 4 and Tables 5 summarize the absorption spectral transition energies and oscillator strength (f).

**Table 4.** The Calculated electronic transition data obtained using TD/DFT/B3LYР/6-311 G fоr аll D-А monomers in the gas

Monomer	transition	λ_max_ nm	In eV	f	MO/character	Orbital Contributions
CB-BCO-CB	S_0_->S_1_	539.59	2.2977	0.3988	HOMO->LUMO	99.2499
	S_0_->S_2_	443.27	2.7970	0.0082	HOMO-1->LUMO	97.4073
	S_0_->S_2_	425.77	2.9120	0.0068	HOMO-2->LUMO	98.8221
CB-BCT-CB	S_0_->S_1_	575.89	2.1529	0.2577	HOMO->LUMO	99.1964
	S_0_->S_2_	466.65	2.6569	0.0091	HOMO-1->LUMO	98.9740
	S_0_->S_2_	444.98	2.7863	0.0037	HOMO-2->LUMO	99.0077
CB-BCS-CB	S_0_->S_1_	577.04	2.1486	0.2515	HOMO-1->LUMO	99.1541
	S_0_->S_2_	462.14	2.6828	0.0106	HOMO-1->LUMO	99.0274
	S_0_->S_2_	441.07	2.8110	0.0027	HOMO-2->LUMO	98.9599
CB-OCP-CB	S_0_->S_1_	607.36	2.0414	0.4301	HOMO->LUMO	99.3486
	S_0_->S_2_	495.25	2.5035	0.0170	HOMO-3->LUMO	7.38355
	S_0_->S_2_				HOMO-2->LUMO	4.63053
					HOMO-1->LUMO	87.1411
		478.53	2.5910	0.0124	HOMO-2->LUMO	94.4432
CB-TCP-CB	S_0_->S_1_	657.65	1.8853	0.3081	HOMO-1->LUMO	99.2048
	S_0_->S_2_	523.42	2.3687	0.0216	HOMO-3->LUMO	4.43007
					HOMO-2->LUMO	4.08636
					HOMO-1->LUMO	90.5831
	S_0_->S_2_	503.93	2.4603	0.0085	HOMO-2->LUMO	95.0324
					HOMO-1->LUMO	4.16680
CB-SCP-CB	S_0_->S_1_	657.07	1.8869	0.3005	HOMO-1->LUMO	99.2133
	S_0_->S_2_	518.56	2.3909	0.0225	HOMO-3->LUMO	3.58905
					HOMO-2->LUMO	5.04412
					HOMO-1->LUMO	90.4458
	S_0_->S_2_	499.17	2.4838	0.0086	HOMO-2->LUMO	94.1027
					HOMO-1->LUMO	5.01051
CB-ODP-CB	S_0_->S_1_	672.78	1.8429	0.4308	HOMO-1->LUMO	99.8397
	S_0_->S_2_	568.73	2.1800	0.0000	HOMO-4->LUMO	99.5658
	S_0_->S_2_	552.32	2.2448	0.0171	HOMO-3->LUMO	13.6963
					HOMO-1->LUMO	85.7238
CB-TDP-CB	S_0_->S_1_	739.67	1.6762	0.3270	HOMO-1->LUMO	99.78324
	S_0_->S_2_	628.61	1.9724	0.0000	HOMO-4->LUMO	99.61097
	S_0_->S_2_	587.03	2.1121	0.0210	HOMO-3->LUMO	11.95507
					HOMO-1->LUMO	87.52174
CB-CDP-CB	S_0_->S_1_	734.31	1.6884	0.3181	HOMO-1->LUMO	99.73804
	S_0_->S_2_	632.86	1.9591	0.0000	HOMO-4->LUMO	99.61944
	S_0_->S_2_	580.41	2.1362	0.0229	HOMO-3->LUMO	11.20538
					HOMO-1->LUMO	88.26156

**Table 5.** The Calculated electronic transition data obtained using TD/DFT/B3LYР/6-311 G fоr аll D-А monomers in the chlorobenzene solvent

Monomer	transition	λ_max_ nm	In eV	f	MO/character	Orbital Contributions
CB-BCO-CB	S_0_->S_1_	586.51	2.1139	0.4086	HOMO->LUMO	99.4360
	S_0_->S_2_	476.45	2.6023	0.0104	HOMO-1->LUMO	98.9093
	S_0_->S_2_	448.55	2.7641	0.0097	HOMO-2->LUMO	99.0556
CB-BCT-CB	S_0_->S_1_	598.82	2.0705	0.2884	HOMO->LUMO	99.3035
	S_0_->S_2_	485.63	2.5531	0.0120	HOMO-1->LUMO	99.2133
	S_0_->S_2_	454.18	2.7298	0.0067	HOMO-2->LUMO	99.0781
CB-BCS-CB	S_0_->S_1_	586.34	2.1145	0.2917	HOMO->LUMO	99.2838
	S_0_->S_2_	472.34	2.6249	0.0143	HOMO-1->LUMO	99.1373
	S_0_->S_2_	442.59	2.8013	0.0055	HOMO-2->LUMO	99.0050
CB-OCP-CB	S_0_->S_1_	668.83	1.8537	0.4833	HOMO->LUMO	99.5940
	S_0_->S_2_	530.04	2.3392	0.0373	HOMO-3->LUMO	4.1478
					HOMO-1->LUMO	95.2421
	S_0_->S_2_	505.82	2.4512	0.0100	HOMO-2->LUMO	97.1395
CB-TCP-CB	S_0_->S_1_	685.31	1.8092	0.3781	HOMO->LUMO	99.4473
	S_0_->S_2_	538.87	2.3008	0.0441	HOMO-1->LUMO	97.3850
	S_0_->S_2_	510.97	2.4264	0.0063	HOMO-2->LUMO	97.1841
CB-SCP-CB	S_0_->S_1_	667.46	1.8576	0.3810	HOMO->LUMO	99.4388
	S_0_->S_2_	523.73	2.3673	0.0469	HOMO-1->LUMO	97.6839
	S_0_->S_2_	496.86	2.4954	0.0057	HOMO-2->LUMO	97.2706
CB-ODP-CB	S_0_->S_1_	737.16	1.6819	0.5273	HOMO->LUMO	99.8511
	S_0_->S_2_	584.71	2.1204	0.0317	HOMO-3->LUMO	9.1233
					HOMO-1->LUMO	90.4244
	S_0_->S_2_	560.22	2.2131	0.0188	HOMO-2->LUMO	99.3683
CB-TDP-CB	S_0_->S_1_	758.65	1.6343	0.4308	HOMO->LUMO	99.7324
	S_0_->S_2_	592.02	2.0943	0.0433	HOMO-3->LUMO	6.1727
					HOMO-1->LUMO	93.3798
	S_0_->S_2_	578.76	2.1422	0.0000	HOMO-4->LUMO	99.5686
CB-CDP-CB	S_0_->S_1_	732.97	1.6915	0.4283	HOMO->LUMO	99.6816
	S_0_->S_2_	574.03	2.1599	0.0489	HOMO-3->LUMO	5.0626
					HOMO-1->LUMO	94.4543
	S_0_->S_2_	573.89	2.1604	0.0000	HOMO-4->LUMO	99.5404

Approximately 70% of the solar photon flux is dispersed in the wavelength range of 380 (3.26 eV) to 900 nm (1.38 eV) [6]. As a result, to maximize the number of excitons created and raise the Jsc, the D/A blend should have a broad and robust absorption in the same region. Because PC60BM (as an acceptor) exhibits poor absorption in this region of the solar spectrum (max = 470 nm), the donor molecule must serve as the primary light absorber. As a result, the photoexcitation properties of the 1–9 were investigated to understand better the physical mechanisms involved in photocurrent generation. In addition, the TDDFT method has been used to predict the electronic transition energies of p-conjugated molecules as a cost-effective method [49].

Tables 4 and Tables 5 show that the excitation energy for all substances investigated is about 2 eV. Furthermore, all monomers had absorption maxima in the 539–739 nm wavelength range in gas and 586–758 nm range in the solvent phase, showing that all molecules only have one band in the visible region (abs > 400 nm). These compounds’ absorption spectra revealed a prominent absorption band attributed to the local transition electron. According to the transition nature, the HOMO LUMO transition is the first singlet excitation in the majority of the compounds. Figures 4 and 5 show the maximum (max) wavelengths for UV-vis absorption spectra of all chemicals simulated in the gas and chlorobenzene solvents.

The geometries of our proposed systems’ absorption spectra appear to be consistent with the usual model for other D-A molecules, which shows an absorption spectrum with a more excellent high-energy absorption band than a weaker low-energy absorption band [50]. D-A-D molecules’ unique absorption spectrum differs from the one broad absorption peak observed in standard p-conjugated polymers such as P3HT [51].

#### 3.4.2. Effect of solvent

Because most chemical activities occur in the solution phase, solvent effects garner a lot of interest. Therefore, the solvent effects are included here to guarantee that the calculations are compatible with the typical conditions. The following section goes into greater detail about the impact of the chemical environment. Excitation energies and maximum absorption quantum chemical computations on the examined molecules were performed in vacuum, in chlorobenzene, to investigate the effect of solvents on the ground state molecular geometry. The orders of magnitude of the spectrum changes range from 9.3 nm to 64.3 nm 46.92 nm for all D-A-D monomers examined.

### 3.5. Density of states (DOS)

The DOS plot is a valuable tool for demonstrating molecular orbitals and their importance to chemical bonding. The results of the DOS plot demonstrate an overlapping population in the molecular orbital. The DOS plot shows the composition of the orbital group that contributes to the molecular orbital. Figure 6 depicts the DOS graphs in the gas phase. The density of localized states has a dramatically rising tendency to arise in the 7.5 to 13 eV range. The graph depicts the orbital properties of various energy ranges. It is the primary contribution of carbon’s s orbital and p essential orbital functions in the frontier molecular orbital. Because electron injection from the excited molecule to the conduction band of the acceptor (PC_60_BM) and subsequent regeneration is allowed in a photovoltaic cell, all of the compounds investigated can be employed as organic solar cell components based on V_OC_ values.

## 4. Conclusions

The B3LYP hybrid functional with 6–31 g (d) basis set is used to theoretically investigate a donor-acceptor-donor (D-A-D) type 3,6-carbazole (CB) paired with different -conjugated benzothiazole derivatives monomer. We also estimated the energy difference between the HOMO and LUMO energies. The optical characteristics of polymers, on the other hand, were modelled using time-dependent DFT at the B3LYP level of theory. The HOMO level of the donor and the LUMO level of the acceptor molecule affect the open-circuit voltage of bulk-heterojunction devices utilizing PCBM as acceptor. When compared to other monomers, the lower Eg of CB-TDP-CB and CB-SDP-CB illustrates the importance of intramolecular charge movement, which causes the ingestion spectra to redshift. This is clearly due to the impact of the electron-contributor unit, which is more solid for 3,6-CB-TDP-CB and 3,6-CB-SDP-CB than for other monomers. All monomers had absorption maxima in the 539–739 nm range in gas and 586–758 nm range in the solvent phase, showing that all molecules contain only one band in the visible region (abs > 400 nm). The morphologies of the absorption spectra of our constructed systems appear to be equivalent to the usual model for other D-A molecules, indicating an absorption spectrum with a greater high-energy absorption band than a weaker low-energy absorption band.
